# Observation of risk factors, clinical manifestations and genetic characterization of recent Newcastle Disease Virus outbreak in West Malaysia

**DOI:** 10.1186/s12917-015-0537-z

**Published:** 2015-08-21

**Authors:** Seetha Jaganathan, Peck Toung Ooi, Lai Yee Phang, Zeenathul Nazariah Binti Allaudin, Lai Siong Yip, Pow Yoon Choo, Ban Keong Lim, Stephane Lemiere, Jean-Christophe Audonnet

**Affiliations:** Department of Clinical Studies, Faculty of Veterinary Medicine, Universiti Putra Malaysia, 43400 UPM, Serdang, Selangor Malaysia; Department of Biotechnology, Faculty of Biotechnology & Molecular Science, Universiti Putra Malaysia, 43400, UPM, Serdang, Selangor Malaysia; Asia-Pacific Special Nutrients Sdn. Bhd, Lot 18B, Jalan 241, Section 51A, 46100 Petaling Jaya, Selangor Malaysia; Vet Food Agro Diagnostic Sdn. Bhd., Lot 18B, Jalan 241, Section 51A, 46100 Petaling Jaya, Selangor Malaysia; Rhone Ma Malaysia (M) Sdn Bhd, Lot 18B, Jalan 241, Section 51A, 46100 Petaling Jaya, Selangor Malaysia; Merial S.A.S., Bio R&D, 254, Rue Marcel Merieux, 69007 Lyon, France

**Keywords:** Newcastle disease virus, Infectious Bursal Disease, Marek’s Disease, Immunosuppressive agents, Recent outbreak, Risk factors, Phylogenetic study, Genetic characterization

## Abstract

**Background:**

Newcastle disease virus remains a constant threat in commercial poultry farms despite intensive vaccination programs. Outbreaks attributed to ND can escalate and spread across farms and states contributing to major economic loss in poultry farms.

**Results:**

Phylogenetic analysis in our study showed that eleven of the samples belonged to genotype VIId. All farms were concurrently positive with two immunosuppressive viruses; Infectious Bursal Disease Virus (IBDV) and Marek’s Disease Virus (MDV). Amino acid sequence analysis confirmed that eleven of the samples had sequence motifs for velogenic/mesogenic strains; three were lentogenic.

**Conclusion:**

In conclusion, no new NDV genotype was isolated from the 2011 NDV outbreak. This study suggests that the presence of other immunosuppressive agents such as IBD and MDV could have contributed to the dysfunction of the immune system of the chickens, causing severe NDV outbreaks in 2011. Risk factors related to biosecurity and farm practices appear to have a significant role in the severity of the disease observed in affected farms.

## Background

Newcastle disease (ND) is a highly contagious viral disease in domestic poultry, aviary and wild birds. Despite intensive vaccination programs, the virus remains a constant threat to the commercial poultry farms in Malaysia [1]. The disease is classified in the World Organization for Animal Health (OIE) as a notifiable disease (formerly list A) [2, 3]. It is a member of the order *Mononegavirales*, family *Paramyxoviridae* and genus *Avulavirus* with an enveloped virus which has a negative-sense, non-segmented single-stranded RNA genome consisting of 15,586 nucleotides [4]. Its genome comprises six genes: nucleoprotein (NP), phosphoprotein (P), matrix protein (M), fusion glycoprotein (F), hemagglutinin-neuraminidase (HN) glycoprotein and large polymerase protein (L). Of the 6 genes found in NDV, its two membrane proteins, the F gene and the HN gene are most important in determination of its virulence. The fusion (F) protein is responsible in mediating fusion of the viral envelope with cellular membranes and the haemagglutinin-neuraminidase (HN) protein is involved in cell attachment and release [4–6].

Newcastle disease virus strains are classified into 3 pathotypes, highly virulent (velogenic), intermediate (mesogenic) or non-virulent (lentogenic) [7]. Traditionally, NDV pathotypes are most commonly distinguished by nucleotide sequencing. The consensus sequence of the F protein cleavage site of velogenic and mesogenic strains is ^112^(R/K)RQ(R/K)RF^117^; the consensus sequence of the lentogenic F cleavage site is ^112^(G/E)(K/R)Q(G/E)RL^117^ [7, 8]. A recent finding documented that a change of glutamine to basic residue arginine (R) at position 114 of the F cleavage site reduced the viral replication and attenuated the virus pathogenicity. The paper also reported that the pathogenicity was further reduced when isoleucine (I) at position 118 was substituted by valine [9].

NDV isolates have been classified into lineages or genotypes based on the analysis of the fusion (F) gene. Aldous et al. 2003 initially used the lineage classification system which grouped NDV isolates into six lineages (1–7) and 13 sub-lineages. Another lineage (lineage 7) and seven other sub-lineages were later proposed. Another classification for NDV classifies the virus into two major groups called class I and class II. There are nine genotypes (1–9) in Class I and eleven genotypes (I-XI) in Class II (2) with genotypes I, II, VI, and VII being further divided into sub-genotypes 1a and 1b, II and IIa, VIa through VIf and VIIa through VIIh [10–13]. Genotype I consists of the avirulent strains of NDV, while viruses of genotypes II, III and IV were reported to be responsible for the first panzootics before the 1960s [10]. Genotype V was thought to be responsible for the second panzootics in the early 1970s and genotype VII viruses caused the third panzootics in racing pigeons during the 1980s [4, 6, 8, 14–21]. Severe outbreaks in Western Europe, South Africa and Southern Europe, Taiwan and China since the early 1990s have been caused by the prevalent genotype VII (VIIa-VIId), constituting the fourth panzootic of NDV [14–16, 18, 22]. Genotype VIII has been found to cause enzootic infections in Southern Africa and is believed to have originated from the Far East [15]. Genotype IX of the ND virus has caused sporadic NDV infections in some regions of China [2, 19], whereas Tsai et al. first demonstrated novel genotype X viruses in Taiwan [18, 21, 23, 24].

The clinical signs and pathological lesions of ND vary with the age and species of birds, the immune status of the host and environmental conditions. A very high number of NDV cases with high mortality in broilers and lower prevalence in layers, breeders and native broilers were reported by field veterinarians towards the end of 2010 through 2011 in Malaysia. Newcastle disease virus infection was suggested as the tentative diagnosis on the basis of history, clinical syndrome, gross lesions, serology as well as isolation of NDV, or the presence of NDV by PCR and/or molecular characterization of the fusion protein gene. These cases were reported in farms which had various combinations of primary and booster vaccinations with lentogenic as well as mesogenic NDV vaccine, or inactivated NDV vaccines. Though vaccinated with NDV vaccines, some of the chicken farms were totally wiped out. Various hypotheses were raised to explain the occurrences of the disease which included 1) a change in pathogenicity of ND virus, 2) vaccine and vaccination practices, and 3) concurrent infection with other immunosuppression organisms or presence of other non-infectious agents. Previous studies have demonstrated the presence of genotype VII and genotype II in Malaysia from 2004 until 2010 [1, 20, 25]. Here we will describe the genetic characterization of the recent isolates from the 2011 NDV outbreaks in West Malaysia with the objective of assessing the risk factors associated with the disease in affected farms. This will investigate the presence of IBDV and MDV as possible immunosuppressive agent, and to determine if there are any new NDV genotype in the 2011 outbreak cases.

## Methods

### Animals ethics

All samples were collected under the supervision of institution veterinarians. The study was conducted following the guidelines as stated in the Code of Practice for Care and use of Animals for Scientific Purposes as stipulated by Universiti Putra Malaysia and complied with the current guidelines for the care and use of animals and was approved by the Institutional Animal Care and Use Committee (IACUC), Faculty of Veterinary Medicine, Universiti Putra Malaysia. There was no experimental research done on the animals. No animals were deliberately sacrificed or injured during the sampling procedure. Every effort was made to minimize any distress or unnecessary culling.

### Sampling

**Fig. 1:**
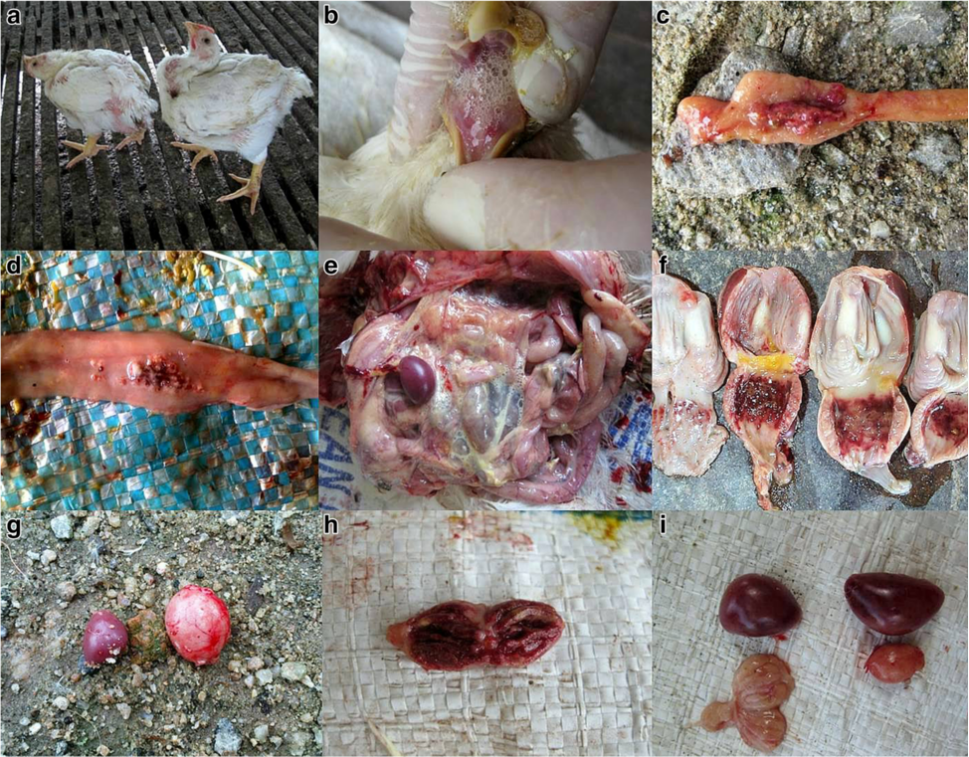
Clinical signs observed from the outbreak. **a** A typical torticollis is shown. These symptoms normally occur 7 to 10 days after a complaint of high mortality is reported. **b** In severely affected birds, mild swollen head and dyspnea with profuse secretions in the trachea were found. **c** & **d** Hemorrhagic & necrosis of intestines especially the caecal tonsils & peyer’s patches were found. **e** Upon PM, the trachea was severely congested and late in the disease stages pericarditis, perihepatitis and caseous air sacculitis were observed. **f** Proventicular hemorrhages were consistent. **g**, **h** & **i** Bursa atrophy was also commonly found in the outbreak. The cut surfaces of the bursa were hemorrhagic – quite atypical from ND infection which prompted us to look for other infectious agents. Not shown above was atrophic thymus

Organ samples were collected from twelve broiler farms displaying typical clinical signs for ND (Fig. 1) in various states in West Malaysia. Five sick birds per farm were sacrificed for the collection of specimens (trachea, lung, spleen, caecal tonsils, proventriculus, intestine, brain, liver, kidney, lymph nodes, bone marrow and bursal of Fabricius). Two farms from East Malaysia with no clinical ND infection were included into the study as negative control.

Consent was obtained from all farm owners for the harvesting of chicken organ samples from their farms.

### Observation of risk factors and clinical manifestation

A standardized survey was used to assess risk factors associated with the high mortality observed. The survey covered the following parameters 1) flock characteristics, 2) vaccination programs, 3) mortality and morbidity rates, 4) age of occurrence, clinical and necropsy lesions and 5) farm management factors such as single/multi age farming practices, source of day old chick (DOC), breed of broilers, number of houses affected, stocking density, disease in neighboring farm, previous history of poor performance/mortality, measures taken in outbreak, measures taken after outbreak for next cycle and performance of the next 2 grow-outs after first incidence. A scoring system (1–5) with 1 (poor) to 5 (very good) was used to evaluate the biosecurity and disinfection status of farms. The data was compiled and analyzed using *t*-test, chi (χ)^2^ and appropriate statistical tests to compare with the negative controls.

### Serology

Serum samples were collected from only four farms from vaccinated flocks (25–40 days), as the study was conducted from commercial production farms with current ND outbreaks. It was not possible to obtain paired serum samples for comparison from every farm as the severity of the outbreak caused very high mortality. The ND-HI titer analysis was conducted by Vet Food Agro Diagnostics (M) Sdn. Bhd.

### Screening of NDV, IBD, MDV

Organ samples were pooled for screening by real-time PCR for NDV and for other immunosuppressive agents i.e., MDV and IBDV. The organ samples were subjected to nucleic acid extraction by using the Trizol LS reagent (Invitrogen, USA) following the standard manufacturer’s protocol. The real time PCR was established from methods previously described [1, 26, 27]. The real time PCR mixtures consisted of 10 μl of SYBR green master mix, the respective primer sets and PCR grade water to make up the final volume of 20 μl per reaction. The PCR mixtures were subjected to real time PCR amplification in a 384-well microplate in the LC480 Real Time PCR instrument (LC 480, Roche). The melting peaks and melting curves were observed by using the Absolute Quant Software provided with the instrument. The respective primer sets are as follow, NDV primers specific for the fusion protein gene, 5′-ATG GGC(C/T) CCA GA(C/T) CTT CTA C-3′ (forward) and 5′-CTG CCA CTG CTA GTT GTG ATA ATC C-3′ (reverse) (Amplicon size: 545 bp); IBD primers, 5′-GT RAC RAT CAC ACT GTT CTC AGC-3′ (Y=C/T); (R=A/G) (forward) and 5′-GAT GTR AYT GGC TGG GTT ATC TC-3′ (reverse) (Amplicon size: 248 bp); MDV-serotype 1 primers, 5′-GAC TCG CTC GCA CAT C-3′ (forward) and 3′-CGA CAC TCC GCA GTT-5′ (reverse) (Amplicon size: 102 bp); MDV-serotype 2 primers; 5′-GTT TCG TCT ACC ACC CG-3′ (forward) and 5′-ATG CCA CTG TAT TTG ATC TCC-3′ (reverse) (Amplicon size: 139 bp); MDV-serotype 3 primers, 5′-ACC GCA ACT CTT CTC ACA-3′ (forward) and 3′-CTC GGG CAA CCT CTA CAT-5′ (reverse) (Amplicon size: 201 bp). Primer sets for MDV serotype 1, 2 and 3 were designed by using the Primer Premier 5 software from Premier Biosoft.

### Sequencing, amino acid sequence analysis, phylogenetic construction of NDV from the 2011 outbreak

The same primer sets for amplifying NDV as described above [1, 26] were used for sequencing. The PCR products of the expected amplicon sizes were purified by using the PCR clean-up gel extraction kit according to the manufacturer’s protocol with slight modifications (Analytik Jena, Germany). Sequencing of the fusion protein gene of NDV from the 14 farms was done in a commercial sequencing facility using the BigDye® Terminator v3.1 cycle sequencing kit. In order to confirm that all positive cases were true Newcastle disease virus, a Basic Local Alignment Search Tool (BLAST) search of the sequence was done in the Genbank® database (Data not shown). The sequence editing and assembly were done by using BioEdit® Sequence Alignment Editor version 7.0.5.2 (Tom Hall, US). Sequences were aligned by using ClustalX™. The phylogenetic tree was constructed by using the distance-based neighbor joining method by using Mega™ 5 software (Biodesign Institute, Tempe, Arizona) and evaluated using the bootstrapping method calculated on 1000 repeats of the alignment. The sequence identity matrix was generated with BioEdit® Sequence Alignment Editor version 7.0.5.2 (Tom Hall, US). All sequences used for constructing the phylogenetic tree are listed in Table 1.

**Table 1:** NDV isolates derived from this study and other isolates reported previously

No	Isolate name	Genbank® accession number	Genotype	Country	Reference
1	V4 Queensland	M24693	I	Australia	Genbank®
2	Ulster/67	M24694	I	N. Ireland	Genbank®
3	F7	JN613118	I	Malaysia	This study
4	Lasota	M24696	II	USA	Genbank®
5	MB061/07	GQ901891	II	Malaysia	Genbank®
6	F5	JN613116	II	Malaysia	Genbank®
7	F6	JN613117	II	Malaysia	Genbank®
8	Miyadera	M24701	III	Japan	Genbank®
9	Italien	EU293914	IV	Italy	Genbank®
10	Herts/33	AY741404	IV	UK	Genbank®
11	CA1085/71	AF001106	V	USA	Genbank®
12	H-10/72	AF001107	V	Hungary	Genbank®
13	TX3503/04	EU477190	VI	USA	Genbank®
14	NDV05-027	DQ439885	VI	China	Genbank®
15	Q-GB 506/97	AF109887	VI	UK	Genbank®
16	DK-1/95	AF001129	VI	Denmark	Genbank®
17	Iraq AG68	AF001108	VI	Iraq	Genbank®
18	Lebanon-70	AF001110	VI	Lebanon	Genbank®
19	MB047/05	GQ901895	VIIa	Malaysia	Genbank®
20	Cockatoo/14698/90	AY288998	VIIa	Indonesia	Genbank®
21	ZA360/95	AF109876	VIIb	S. Africa	Genbank®
22	ZW3422/95	AF109877	VIIb	Zimbabwe	Genbank®
23	NDV05-055	DQ439910	VIIc	China	Genbank®
24	TW/2000	AF358786	VIIc/d	Taiwan	Genbank®
25	F8	JN613119	VIId	Malaysia	Genbank®
26	Ch/2000	AF358788	VIId	China	Genbank®
27	MB064/05	GQ901893	VIId	Malaysia	Genbank®
28	MB016/07	GQ901894	VIId	Malaysia	Genbank®
29	F1	JN613112	VIId	Malaysia	This study
30	F2	JN613113	VIId	Malaysia	This study
31	F3	JN613114	VIId	Malaysia	This study
32	F4	JN613115	VIId	Malaysia	This study
33	F9	JN613120	VIId	Malaysia	This study
34	F10	JN613121	VIId	Malaysia	This study
35	F11	JN613122	VIId	Malaysia	This study
36	F12	JN613123	VIId	Malaysia	This study
37	F13	JN613124	VIId	Malaysia	This study
38	F14	JN613125	VIId	Malaysia	This study
39	MB043/06	GQ901896	VIId	Malaysia	Genbank®
40	MB091/05	FJ008916	VIId	Malaysia	Genbank®
41	MB093/05	FJ008917	VIId	Malaysia	Genbank®
42	MB095/05	FJ008918	VIId	Malaysia	Genbank®
43	MB128/04	FJ008923	VIId	Malaysia	Genbank®
44	DE143/95	AF109881	VIId	UK	Genbank®
45	TW/95-1	AF083960	VIIe	Taiwan	Genbank®
46	MB076/05	GQ901892	VIIe	Malaysia	Genbank®
47	AF2240	AF048763	VIII	Malaysia	Genbank®
48	MB085/05	GQ901901	VIII	Malaysia	Genbank®
49	QH-1/79	AF378250	VIII	China	Genbank®
50	QH-4/85	AF378252	VIII	Malaysia	Genbank®
51	ZhJ-1/85	AF458023	IX	Malaysia	Genbank®
52	FJ-1/85	AF458009	IX	Malaysia	Genbank®

## Results & discussion

### Observation of risk factors and clinical manifestations

The results of the survey are shown in Tables 2, 3, 4 and 5. Table 2 describes farm characteristics and type of breeds used. All farms were multi-age broiler farms stocked with various commercial breeds namely Cobb, Ross or commercial native birds. The mean flock population was about 90,000 broilers (83.3 % of houses were open sided houses). On average, 7.2 houses were affected. Over 90 % of the farms surveyed were located within 1 km radius to other broiler and reported high mortality in neighboring farms. More than 50 % of the farms had poor performance in its two previous grow-outs (*P* < 0.05), reported that the day-old-chick quality were poor in the affected flock (*P* < 0.01) and had unsubstantiated evidence that the chicks were sourced from breeder flocks which had been reported with high mortalities resembling ND (*P* < 0.05). The average age of onset of disease was 15.9 (*P* < 0.05) days old which was reported to be at the time or after IBD vaccinations were administered and 28 days in the negative control farms.

**Table 2:** Comparison of serology titer from the surveyed ND outbreak vs. serology titer from non-survey farms ND outbreak

		GMT	Mean
Farm 6 (survey)	ND outbreak with IBD and MD detected	1.5	1.8
Farm 4, 7, 8 (survey)	ND outbreak with IBD and MD detected	212.8	390.4

**Table 3:** Farm characteristics and history

Risk factor	Affected farms	Negative controls
No of farms	12	2
Number of broilers per farm	89,950^a^	87,500^a^
Number of houses per farm	11.1^a^	6.5^a^
Number of houses affected per farm	7.2^a^	1.5^a^
Type of management – multi-age	Multi-age	Multi-age
Type of housing – open-sided housing	Open	Open
Farms within 1 km of affected farm	92 %^a^	50 %^a^
Poor performance in the last 2 grow outs	50 %^c^	0 %^d^
Neighboring farms with history of disease	91.7 %^a^	0 %^b^
Complaints of poor day-old-chick quality	50 %^c^	50 %^d^
Disease after IBD vaccination or about Week 2-3	83.3 %^a^	50 %^b^
Suspected source of chicks from breeder farms with disease	66.7 %^a^	0 %^b^

Note: ^a,b^Values in different columns bearing different superscripts are significantly different (*P* < 0.05), ^c,d^Values in different columns bearing different superscripts are significantly different (*P* < 0.01)

**Table 4:** Vaccination and assessment of biosecurity and sanitation status

Risk factor	Affected farms	Negative controls
Biosecurity status	1.7^a^	2.5^a^
Sanitation and disinfection status	2.6^a^	2.5^a^
Vaccination with NDv	100 %^a^	100 %^a^
Vaccination with IBv	100 %^a^	100 %^a^
Vaccination with IBDv	91.7 %^a^	91.7 %^a^
Vaccination with MDv	8.3 %^a^	0 %^b^
Vaccination with SHSv	8.3 %^a^	0 %^b^

Note: ^a,b^Values in different columns bearing different superscripts are significantly different (*P* < 0.05), ^c,d^Values in different columns bearing different superscripts are significantly different (*P* < 0.01)

**Table 5:** Zootechnical results, clinical and necropsy findings

Risk factor	Affected farms	Negative controls
Age of first occurrence of disease	15.9^a^	28.0^a^
Age of clinical and necropsy examination	24.8^a^	32.5^a^
Mortality at grow-out, %	32.3 %^a^	4.5 %^b^
Presence of respiratory disease	100 %^a^	100 %^a^
Presence of enteric disease	100 %^a^	0 %^b^
Torticolis and neurological signs	91.7 %^a^	0 %^b^
Haemorrhages in more than 1 visceral organ	83.3 %^a^	0 %^b^
Thymus atrophy	100 %^a^	0 %^b^
Bursal atrophy	75 %^a^	50 %^b^
Ascites	8.3 %^a^	50 %^a^
Air-sacculitis, perihepatitis and peritonitis	16.7 %^a^	50 %^b^
Clouded air sacs	50 %^c^	100 %^d^

Note: ^a,b^Values in different columns bearing different superscripts are significantly different (*P* < 0.05), ^c,d^Values in different columns bearing different superscripts are significantly different (*P* < 0.01)

The frequency of vaccination in the 12 study farms for Newcastle disease, Infectious Bronchitis, Infectious Bursal disease, Marek’s disease and Swollen Head Syndrome (SHS); a disease caused by Avian metapneumovirus; were 100 %, 100 %, 91.7 %, 8.3 % and 8.3 % respectively (Table 4). The frequency of ND vaccination for lentogenic, mesogenic and inactivated vaccines were 100 %, 33.3 % and 50.0 % with all farms practicing multiple ND vaccinations in the lifecycle of the broilers. Biosecurity and farm sanitation scores were 1.7 and 2.6 respectively.

The onset of clinical ‘ND’ disease was reported at 15.9 days old (*P* < 0.05) with a final grow out mortality of affected flocks at 32.3 % (*P* < 0.05) (Table 5). All the farms initiated treatment with antimicrobials and supportive treatments (vitamins and electrolyte) and upgraded sanitation and disinfection practices when the disease was observed or when advised by field veterinarians. The primary clinical signs were inappetance, depression, ruffled feathers, whitish to greenish and watery diarrheoa, head swelling and dyspnoea. Torticollis was seen at about 7–10 days after the onset of clinical signs. Gross pathology lesions included profuse fluids in the trachea and bronchus, congested trachea, gizzard and proventricular erosions and hemorrhages, hemorrhages on Peyer’s patches and caecal tonsils and in other visceral organs. Bursa (*P* < 0.05) and thymus atrophy were present in the majority of cases. The disease process appeared to be of acute onset with significantly reduced observations of air sacculitis, perihepatitis and peritonitis (*P* < 0.05). NDV, IBDV and MDV were detected in organs and tissues at 100 %, 83 % and 83 % respectively by PCR, with MD Serotypes 2, 1 and 3 in descending order of detection. NDV, IBDV and MDV were detected concurrently in 75 % of farms (Table 6). The concurrent presence of IBDV and MDV with NDV were significant at *P* < 0.05 and with the presence of MDV Serotype 1 (*P* < 0.01).

**Table 6:** Detection of infectious agents concurrently with NDV positive samples

Presence of concurrent infectious agent in disease farms	Frequency of detection
IBD	83.0 %
MD	83.0 %
IBD+MD	75.0 %
MD Serotype 1	58.0 %
MD Serotype 2	75.0 %
MD Serotype 3	67.0 %

### Serology

Serology results from one farm (Farm 6) showed that the antibody titer was low and not as expected with a mean titer of 1.8. Antibody titer from three other farms (Farm 4, 7 and 8) was very high for a broiler which indicates that there’s an infection.

### Screening for NDV, IBD and MDV

Samples from all farms were found to be positive for NDV, IBD and MDV, and clearly evident that immunosupression continues to be a major problem for the poultry industry. IBDV is one of the most common immunosuppressive agents in poultry. It primarily targets the bursa of Fabricious which is committed to the differentiation and proliferation of B-lymphocytes into antibody-producing plasma cells. In the bursa, IBDV produces severe destruction of B-lymphocytes by either necrosis or apoptosis, and consequently the antibody-mediated response (humoral) is affected [27, 28]. Recent studies have also demonstrated that this virus can also hinder some of the mechanisms of cellular immunity making chickens more susceptible to viral respiratory infections and elevating mortality. Thus, the immune response to vaccines is impaired and the overall productive performance may be significantly decreased in all types of chickens. Similarly, Marek’s Disease, which is an ubiquitous, complex, lymphoproliferative disease of chickens caused by a strong cell-associated alpha herpesvirus, MD virus (MDV) is progressive in nature with a relatively long incubation period and the virulent virus can remain in the host without producing any clinical syndromes [23, 24, 29, 30]. The primary cell targets of MDV infection are lymphocytes, and as a result, early effects are mainly seen in lymphoid organs such as the bursa of Fabricius (source of B lymphocytes), the thymus (primary source of T lymphocytes) and the spleen. Consequences of these MDV-induced immunosuppresion have also shown to cause reduced resistance to other concurrent infections [29–31]. Therefore, based on the findings, the presence of IBD and MDV suggest a significant negative impact on the immune system and growth of these broiler chickens. NDV itself was not immunosuppressive, and vaccination with killed-NDV vaccines failed to reduce incidences of NDV outbreaks, because the core underlying factors such as IBD and MDV that were causing sub-clinical infections were not addressed, thus reducing the protective effect of the NDV vaccination programs.

### Sequencing, amino acid sequence analysis, phylogenetic construction

**Fig. 2:**
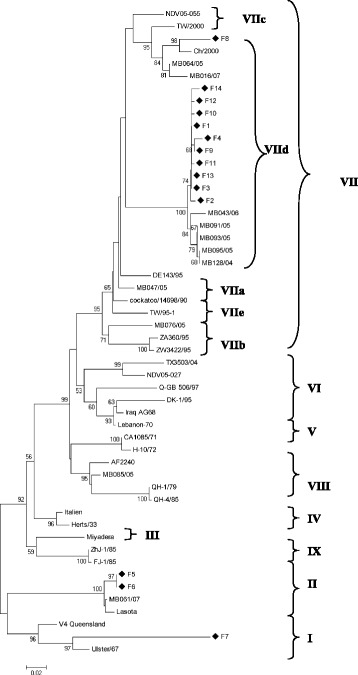
Phylogenetic Tree constructed with 52 NDV sequences obtained from this study and Genbank^®^. The tree was designed by using the neighbour-joining method on Mega 5. The sequences from this study are indicated by the diamond shaped symbol

BLAST® analysis showed that all samples were true NDV cases when compared with other sequences on Genbank®. Amino acid sequence analysis of the 535 bp fragment of the fusion protein (F) gene of the fourteen Malaysian NDV isolates showed that eleven of the isolates were categorized as velogenic virus and three were lentogenic. The eleven velogenic strain had the F cleavage site motif ^112^R-R-R-K-R-F^117^ while two of the lentogenic strain had the F cleavage site motif ^112^G-R-Q-G-R-L^117^, whilst one sequence had the F cleavage site motif ^112^G-K-Q-G-R-L^117^ at the C-terminus of the F2 protein and phenylalanine (F) residue at amino acid position 117 of the N-terminus of the F1 protein (Table 7). Phylogenetic analysis revealed that 11 of the Malaysian isolates clustered closely with the genotype VIId strains, one of the Malaysian isolate grouped together with genotype I and two of the isolates grouped with genotype II (Fig. 2). Of the eleven Malaysian isolates that grouped to form genotype VIId, ten (F1, F2, F3, F4, F9, F10, F11, F12, F13, F14) had between 97.9 to 98.7 % sequence identity similarities with other Malaysian isolates responsible for the Newcastle disease outbreaks in 2004–2005 and 2007 which were previously reported by researchers from Universiti Putra Malaysia [20, 28, 31]. All these isolates have between 91 and 92 % similarities with the Indonesian strain (cockatoo/14698/90). One (F8) of the Malaysian isolate which grouped with genotype VIId had 96.1 % similarities with the China strain (Ch/2000). Of the three lentogenic strains isolated from this study, two (F5, F6) had between 97.4 and 97.5 % nucleotide sequence similarities with genotype II while one isolate (F7) had around 88.8 % nucleotide sequence similarities with genotype I.

**Table 7:** The F cleavage site and it’s pathotypes from the Malaysian isolates

Isolate	Genbank® accession no	F cleavage site	Genotype	Pathotype	Source
F1	JN613112	RRRKRF	VIId	Velogenic/Mesogenic	This study
F2	JN613113	RRRKRF	VIId	Velogenic/Mesogenic	This study
F3	JN613114	RRRKRF	VIId	Velogenic/Mesogenic	This study
F4	JN613115	RRRKRF	VIId	Velogenic/Mesogenic	This study
F5	JN613116	GRQGRL	II	Lentogenic	This study
F6	JN613117	GRQGRL	II	Lentogenic	This study
F7	JN613118	GKQGRL	I	Lentogenic	This study
F8	JN613119	RRRKRF	VIId	Velogenic/Mesogenic	This study
F9	JN613120	RRRKRF	VIId	Velogenic/Mesogenic	This study
F10	JN613121	RRRKRF	VIId	Velogenic/Mesogenic	This study
F11	JN613122	RRRKRF	VIId	Velogenic/Mesogenic	This study
F12	JN613123	RRRKRF	VIId	Velogenic/Mesogenic	This study
F13	JN613124	RRRKRF	VIId	Velogenic/Mesogenic	This study
F14	JN613125	RRRKRF	VIId	Velogenic/Mesogenic	This study

Overall, based on the amino acid sequence analysis, eleven of the farms displayed the typical sequence motifs for velogenic/mesogenic, while three had the sequence motif for lentogenic strains. This correlates with the fact that two of the samples (F6 & F7) were collected as negative controls from farms with no mortality. Based on phylogenetic investigations, eleven (F1, F2, F3, F4, F8, F9, F10, F11, F12, F13, F14) of the samples clustered with the genotype VIId and had a very close relationship with the previously isolated Malaysian isolates (MB043/06, MB091/06, MB093/05, MB095/05, MB128/04) which suggest that similar NDV strains have been circulating in this region for several years now [1, 20, 25].

### Nucleotide sequence accession numbers

The complete genomic sequences of the 14 NDV isolates reported in this paper were deposited with the GenBank® database under accession numbers JN613112, JN613113, JN613114, JN613115, JN613116, JN613117, JN613118, JN613119, JN613120, JN613121, JN613122, JN613123, JN613124 and JN613125.

These sequences are downloadable from Entrez™ Pop Set data as a group of sequences.

## Conclusion

The present study confirms that similar velogenic NDV genotype VIId, as reported previously, were detected in the study farms in spite of vaccination. In addition, no new genotype of ND virus was found based on the genetic characterization. IBDV and MDV were also concurrently detected by PCR. Immunosuppressive agents play a significant role in vaccination failures. However, the role, interactions and effect of these immunosuppressive agents as well as other concurrent infectious and non infectious agents not studied in the disease process could not be fully ascertained. Risk factors such as multi-age production practices, close proximity of farms, biosecurity and sanitation practices appear to have a role in the outcome of the disease, in terms of severity, mortality, clinical and pathological findings. Preventive measures taken post outbreak such as improved biosecurity and sanitation appear to have mediated improved performance in subsequent grow-outs. The findings from this study suggest that there may not be a need for a new vaccine as the same genotype that has been present for a long time is still responsible for the current ND outbreaks. Further to that, the findings also suggest that risk factors related to biosecurity and farm practices appear to have a significant role in the severity of the disease observed in affected farms. If those factors are alleviated, the severity of the ND problems in farms would be greatly reduced.

## Abbreviations

NDV: Newcastle disease virus
IBDV: Infectious bursal disease virus
MDV: Marek’s disease virus
PCR: polymerase chain reaction
MDV: Marek’s Disease Virus
SHS: Swollen Head Syndrome
